# Resonance Raman analysis of intracellular vitamin B_12_ analogs in methanogenic archaea

**DOI:** 10.1002/ansa.202100042

**Published:** 2022-01-09

**Authors:** Nanako Kanno, Shingo Kato, Takashi Itoh, Moriya Ohkuma, Shinsuke Shigeto

**Affiliations:** ^1^ Department of Chemistry School of Science Kwansei Gakuin University Sanda Hyogo Japan; ^2^ Japan Collection of Microorganisms RIKEN BioResource Research Center Tsukuba Ibaraki Japan

**Keywords:** cobamides, methanogens, nondestructive cell analysis, resonance Raman spectroscopy

## Abstract

Methanogenic archaea (methanogens) are microorganisms that can synthesize methane. They are found in diverse environments ranging from paddy fields to animal digestive tracts to deep‐sea hydrothermal vents. Investigating their distribution and physiological activity is crucial for the detailed analysis of the dynamics of greenhouse gas generation and the search for the environmental limits of life. In methanogens, cobamide cofactors (vitamin B_12_ analogs) play a key role in methane synthesis and carbon fixation, thus serving as a marker compound that metabolically characterizes them. Here, we report on resonance Raman detection of cobamides in methanogenic cells without destroying cells and provide structural insights into those cobamides. We succeeded in detecting cobamides in four representative methanogens *Methanosarcina mazei*, *Methanosarcina barkeri*, *Methanopyrus kandleri*, and *Methanocaldococcus jannaschii*. The former two are mesophilic, cytochrome‐containing methanogens, whereas the latter two are hyperthermophilic, non‐cytochrome‐containing methanogens. The 532 nm‐excited Raman spectra of single or multiple cells of the four species all showed resonance Raman bands of cobamides arising mainly from the corrin ring, with the most intense one at ∼1500 cm^−1^. We envision that resonance Raman microspectroscopy could be useful for in situ, nondestructive identification of methanogenic cells that produce high levels of cobamides.

## 1 INTRODUCTION

Methanogenic archaea (or simply methanogens) are microorganisms that are almost exclusively^1^ known to produce methane and are found in a wide range of anaerobic habitats, such as hydrothermal vents, paddy fields, gastrointestinal tracts of animals, and industrial microbial communities.^2^ Biological methane production has both positive and negative impacts on the ecosystem and our society. Methanogens can be used in anaerobic digestion to break down organic matter and to convert waste to biogas.^3^ On the other hand, significant amounts of methane are regularly emitted from wetlands, rice fields, livestock farms, and the ocean, and contribute to the greenhouse effect.^4^ More fundamentally, because methanogens interact with diverse anaerobic microbial species, methanogenic microbial communities serve as a good model system for investigating the microbial interactions and their ecological consequences.^5^, ^6^ In addition, methanogens have attracted much attention for their relevance to understanding various extreme environments and exploring the environmental limits of life.^7^, ^8^ Therefore, it is of great importance to study the in situ distribution and activity of methanogens from a multitude of perspectives including astrobiology and biotechnology.

**FIGURE 1:**
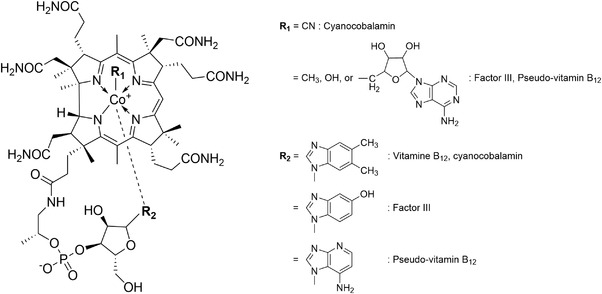
Chemical structures of cobamides (vitamin B_12_, factor III, pseudo‐vitamin B_12_, and cyanocobalamin). Putative in vivo forms of factor III and pseudo‐vitamin B_12_ have the methyl, hydroxyl, or adenosyl upper ligand, which tends to be converted to the cyano group when extracted

One of the physiologically active substances characteristic of methanogens is cobamide cofactors. The most well‐known cobamide is arguably vitamin B_12_, which is essential for human health.^9^, ^10^ Cobamides are involved in the physiological activities of various organisms from animals to prokaryotes including methanogens. In natural microbial communities, the cobamides that are produced by a subset of the microbial population have been shown to be important in nutrient interactions such as cross‐feeding.^11^, ^12^ As shown in Figure 1, cobamides are composed of the cobalt‐containing corrinoid coordinated with upper and lower axial ligands. Because of this extraordinarily complex structure, industrial production of vitamin B_12_ currently relies on fermentation processes using mainly *Propionibacterium* (propionic acid‐producing bacteria) rather than using chemical synthesis.^13^, ^14^ In methanogens, cobamides occur as factor III and pseudo‐vitamin B_12_.^15^ These molecules differ from vitamin B_12_ by the lower axial ligand, where 5,6‐dimethylbenzimidazole in vitamin B_12_ is replaced by 5‐hydroxybenzimidazole in factor III and by adenine in pseudo‐vitamin B_12_ (Figure 1). It has been reported that these cobamides play a key role in methane synthesis and carbon‐fixing reactions in methanogens^14^, ^16^ and that their abundances vary depending on the methanogenic species and the type of substrate for methanogenesis.^17^ Given their ubiquity and significance, in situ analysis with cobamides as a marker compound could be useful for evaluating the distribution and activity of methanogens. However, there has been no report on the detection and analysis of cobamides in methanogenic cells.

In recent years, Raman microspectroscopy has proven itself useful for analyzing microbial cellular activity in environmental samples^18^, ^19^, ^20^ and for discriminating microbial species at the single‐cell level.^21^, ^22^ Here, we demonstrate that resonance Raman microspectroscopy can selectively detect intracellular cobamides in methanogenic cells. We are able to detect cobamides in four methanogenic species: *Methanosarcina mazei*, *Methanosarcina barkeri*, *Methanopyrus kandleri*, and *Methanocaldococcus jannaschii*. *M. mazei* was originally isolated from paddy soil^23^ and *M. barkeri* from an anaerobic sewage–sludge digester.^24^ In contrast, *M. kandleri* and *M. jannaschii* were isolated from a hydrothermally heated deep‐sea sediment^25^ and a submarine hydrothermal vent,^26^ respectively. We observe the resonance Raman bands that change in intensity with laser irradiation time due to photobleaching and attribute those bands to cobamides by comparison with the reference spectrum of cyanocobalamin. Despite a number of in vitro resonance Raman studies on vitamin B_12_ derivatives,^27^, ^28^, ^29^, ^30^, ^31^, ^32^ insights into their structure have not been gained from methanogenic cells. Sensitive in situ detection of cobamides will allow us to identify methanogens that produce high concentrations of cobamides in environmental samples.

## 2 MATERIALS AND METHODS

### 2.1 Reagents

Cyanocobalamin (special grade) was purchased from FUJIFILM Wako Pure Chemical Corporation, and 0.05% (w/v) cyanocobalamin solution in Milli‐Q water was prepared. A 200 μL portion of the aqueous solution was transferred to a glass bottom dish for Raman measurements.

### 2.2 Microbial strains and cell sample preparation

*Methanosarcina mazei* JCM9314, *Methanosarcina barkeri* JCM 10043^T^, *Methanopyrus kandleri* JCM9639^T^, and *Methanocaldococcus jannaschii* JCM 10045^T^ were obtained from the Japan Collection of Microorganisms (JCM). Cell cultures were used as received from JCM for Raman measurements within 7 days of shipping. Cells of each strain except for *M. jannaschii* were washed three times with phosphate buffer solution (PBS) at pH 7.4 by centrifugation (8000–10,000 × *g*, 30 s) at room temperature and subsequent resuspension in PBS. Cells of *M. jannaschii* were washed three times with PBS containing 3% NaCl by centrifugation (10,000 × *g*, 1 min) at 4°C and then resuspended in PBS with 3% NaCl. The cell suspension was diluted for better microscopic observation of individual cells and 200 μL of the cell suspension of each strain was transferred to a glass bottom dish.

### 2.3 Confocal Raman microspectroscopy

The Raman spectra of methanogenic cells and cyanocobalamin were measured using a laboratory‐built confocal Raman microspectrometer, which has been described previously.^33^ The output at ∼532 nm of a continuous‐wave semiconductor laser (Coherent, Genesis CX532‐2000SLM‐CDRH) was used as the Raman excitation light. The beam was focused onto the sample with an oil‐immersion objective lens (Olympus, 100×, NA 1.4, UPLSAPO100X for *M. mazei*, *M. barkeri*, and cyanocobalamin; Olympus, 100×, NA 1.3, UPLFLN100XO2PH for *M. kandleri* and *M. jannaschii*). The laser power at the sample was adjusted to be 3, 2, 1.5, 0.5, and 1 mW for *M. mazei, M. barkeri*, *M. kandleri*, *M. jannaschii*, and cyanocobalamin, respectively.

**FIGURE 2:**
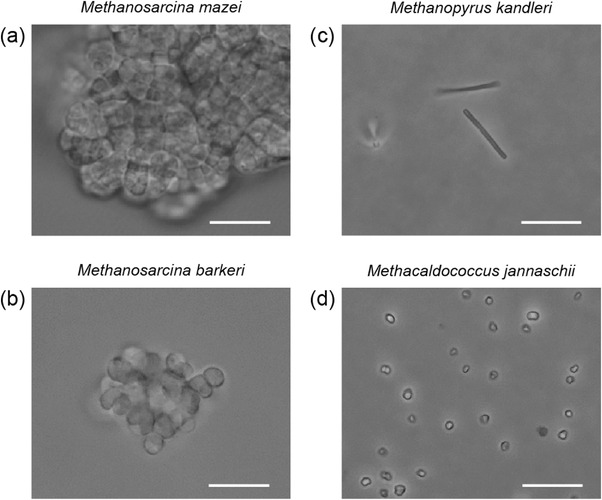
Optical micrographs of *M. mazei* (a), *M. barkeri* (b), *M. kandleri* (c), and *M. jannaschii* (d) cells. Scale bar = 10 μm

*M. mazei* cells, each of which has an irregular coccoid shape with a 1–3 μm diameter,^23^ exhibited multicellular aggregates embedded in a methanochondroitin matrix (Figure 2a). *M. barkeri* cells formed similar aggregates, albeit a little smaller (Figure 2b). To avoid, as much as possible, measuring spectra from multiple cells stacked vertically, the laser beam was focused on the centre of a cell at the edge of an aggregate. Series of Raman spectra of *M. mazei* and *M. barkeri* were measured every 30 s up to 300 s using 30 s exposure time and every 15 s up to 150 s using 15 s exposure time, respectively. In contrast, single long rod‐shaped *M. kandleri* cells (Figure 2c) suspended in PBS were optically trapped at their edge by the same laser beam as that for Raman excitation. The Raman spectra of *M. kandleri* cells were measured every 10 s up to 30 s using 10 s exposure time. Single irregular coccoid‐shaped *M. jannaschii* cells (Figure 2d) suspended in PBS with 3% NaCl were also picked via laser trapping and their Raman spectra were acquired every 15 s up to 75 s using 15 s exposure time. The cells to be measured were randomly selected by visual inspection. The Raman spectrum of cyanocobalamin dissolved in Milli‐Q water was measured with a 60 s exposure time.

Backward scattering was collected by the same objective lens as that used to focus the incident laser light. After passing through three notch filters for Rayleigh scattering rejection and a 100 μm pinhole for confocal detection, the scattered light was analyzed by an imaging spectrometer (SOL Instruments, MS3504i) equipped with a 1200 grooves/mm grating and detected by a thermoelectrically cooled charge‐coupled device (CCD) detector (Andor, DU401A‐BV) with 1024×127 pixels operating at −65°C. The spectral resolution of our spectrometer was estimated to be ∼3 cm^−1^.

### 2.4 Electronic absorption spectroscopy

The UV/Vis absorption spectrum of 0.05% (w/v) aqueous solution of cyanocobalamin was measured with a JASCO V‐750 spectrophotometer.

### 2.5 Data analysis

For *M. mazei* and *M. kandleri*, the PBS spectrum (average of 10 spectra) was subtracted from each single‐cell spectrum in which cosmic rays, if any, were manually removed in advance. For *M. barkeri*, the PBS spectrum with the contribution of the glass substrate was subtracted. The resulting Raman spectra were then baseline‐corrected using fifth‐order polynomial fitting. A fifth‐order polynomial was most effective, with minimal fitting parameters, in eliminating the slowly changing baseline that might arise from cellular autofluorescence and/or the fluorescence of the anaerobic indicator resazurin. Because the Raman spectra of *M. jannaschii* cells showed high noise levels due to low laser power and short exposure time, singular value decomposition (SVD)‐based denoising^34^, ^35^, ^36^ was performed. Negligibly small SV components were discarded as noise, and the remaining components (10 components in the present study) were used to reconstruct noise‐reduced data. Subtraction of the PBS spectrum was not performed for *M. jannaschii*; only baseline correction using a fourth‐order polynomial was performed. The water spectrum (average of 10 spectra) was subtracted from the spectrum of the aqueous solution of cyanocobalamin and its baseline was corrected using third‐order polynomial fitting. In this case, a third‐order polynomial was sufficient to account for the baseline. All of the above preprocessing was performed on Igor Pro 8.04 software (WaveMetrics).

## 3 RESULTS

### 3.1 Raman spectral signatures of methanogenic cells

**FIGURE 3:**
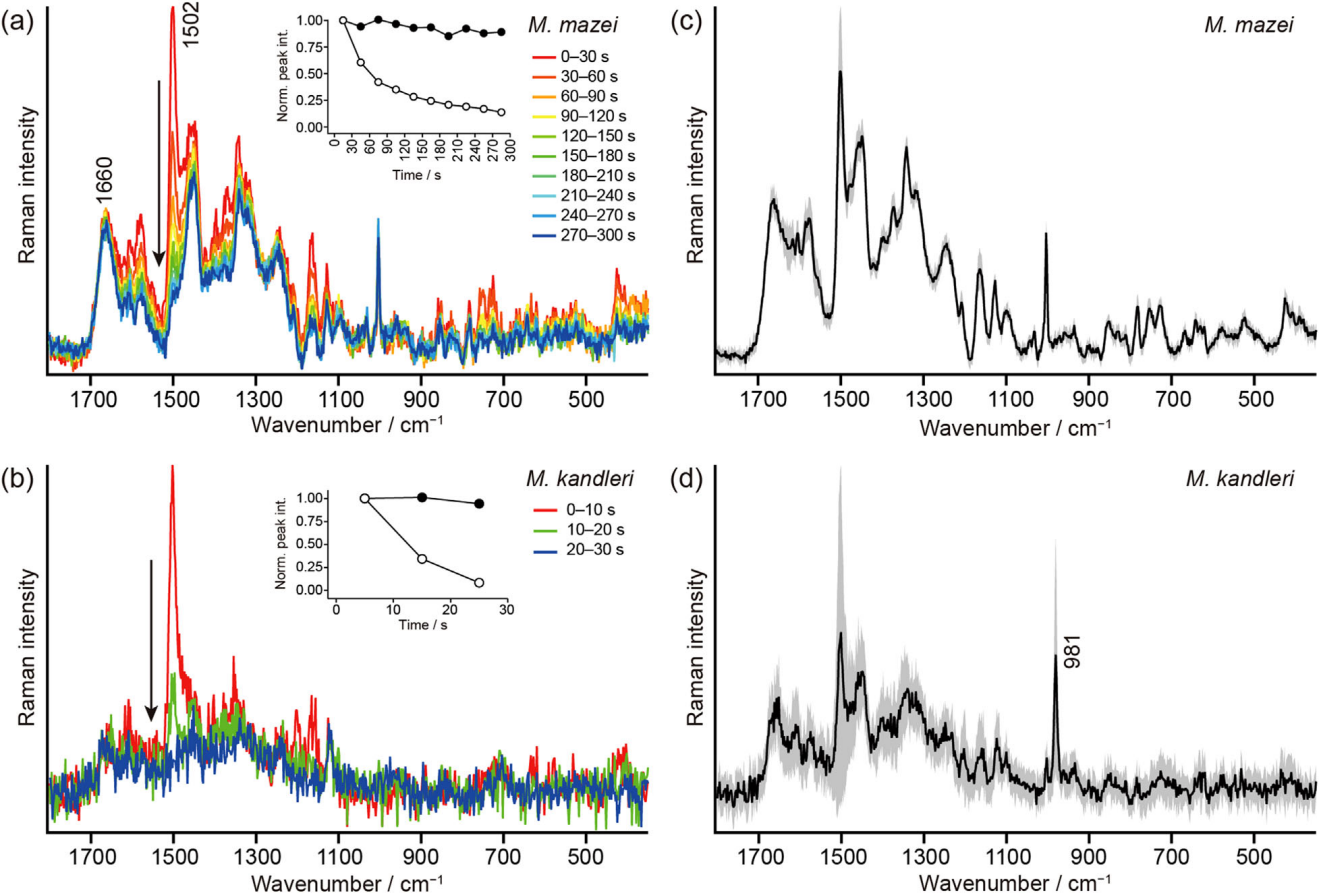
(a) Raman spectra measured within a *M. mazei* cell aggregate every 30 s up to 300 s. The inset shows the peak heights of the 1502 (open circles) and 1660 cm^−1^ (closed circles) bands plotted as a function of the laser irradiation time. The peak height was calculated by averaging the intensities at three consecutive wavenumbers including the peak for both bands. The value of the peak height representing each time interval is displayed at the centre of the interval (e.g., 15 s for 0–30 s). (b) Raman spectra measured within a single *M. kandleri* cell every 10 s up to 30 s. The inset shows the peak heights of the 1502 (open circles) and 1660 cm^−1^ (closed circles) bands plotted as a function of the laser irradiation time. The peak height was calculated by averaging the intensities at 3 (1502 cm^−1^ band) and 21 (1660 cm^−1^) consecutive wavenumbers including the peak. The value of the peak height representing each time interval is displayed at the centre of the interval. (c) Average of the 0–30 s Raman spectra of 10 different *M. mazei* cell aggregates. The shaded area represents the 1σ error envelope. (d) Average of the 0–10 s Raman spectra of 20 different *M. kandleri* cells. The shaded area represents the 1σ error envelope

We first focus on the results of *M. mazei* and *M. kandleri*. Figure 3a shows a representative time‐series of the 532 nm‐excited Raman spectra of *M. mazei* cells in an aggregate. Because the focal spot size of the laser (∼1 μm) was comparable to the cell size (1–3 μm in diameter^23^; Figure 3a), it is likely that these spectra were recorded from multiple *M. mazei* cells rather than a single cell. As the laser irradiation time increases, several Raman bands at, for example, 425, 728, 1165, and 1502 cm^−1^ decrease drastically in intensity, whereas others (e.g., 1003 and 1660 cm^−1^) arising from proteins and nucleic acids remain almost unchanged. The temporal profiles of the intensities of the 1502 and 1660 cm^−1^ bands are plotted in the inset of Figure 3a. This decaying behavior is characteristic of laser‐induced photobleaching of the resonance Raman bands of molecular species whose electronic transition is in resonance with the excitation light. A similar decay of the Raman signatures is also observed for *M. kandleri* (Figure 3b), although the signal‐to‐noise ratio (S/N) of the spectra is somewhat lower and the photobleaching occurs much faster (completed within 30 s) than for *M. mazei*. The lower S/N for *M. kandleri* results from the fact that for *M. kandleri*, what we measured was assuredly a single cell and its size was smaller than that of *M. mazei* (see Figure 2).

Figure 3c,d shows the average of 10 Raman spectra of *M. mazei* and 20 Raman spectra of *M. kandleri* (i.e., 20 single cells), respectively. We observe a large variation in the intensity of the 1502 cm^−1^ band for *M. kandleri*. This result may be due in part to the slight difference among the measured cells in the start time of spectral acquisition after turning on laser irradiation. Therefore, special attention must be paid to the laser power and exposure time, as the ability to detect the bleaching signal depends strongly on these parameters. In addition to the above technical reason, given a similar variation in the intensity of the non‐photobleaching 981 cm^−1^ band of the sulfate ion^37^ observed in *M. kandleri* (Figure 3d), there seems to be an inherent, marked cell‐to‐cell variation within the *M. kandleri* cell population.

### 3.2 Identification of cobamide resonance Raman spectra of methanogens

**FIGURE 4:**
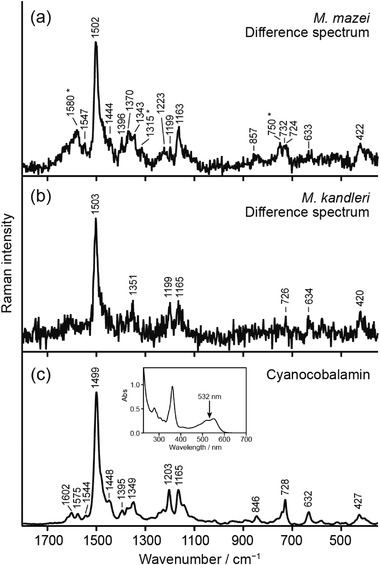
Comparison of the difference Raman spectra of *M. mazei* (a) and *M. kandleri* (b) with the resonance Raman spectrum of cyanocobalamine (0.05 w/v%) in aqueous solution (c). The difference spectra were obtained by subtracting the last (270–300 s for *M. mazei* and 20–30 s for *M. kandleri*) spectrum from the first (0–30 s for *M. mazei* and 0–10 s for *M. kandleri*) spectrum recorded in consecutive measurements (see Figure 3a,b). The cyanocobalamin spectrum is the average of three acquisitions. In panel (a), asterisks indicate Raman bands that originate from cytochromes. The inset of panel (c) displays the UV–Vis spectrum of the cyanocobalamin sample

To identify the molecular species that give rise to the observed resonance Raman bands, the difference spectra were calculated by subtracting the last spectrum (270–300 s for *M. Mazei* and 20–30 s for *M. kandleri*) from the first spectrum (0–30 s for *M. mazei* and 0–10 s for *M. kandleri*) recorded in the time‐series. The difference spectra so obtained of *M. mazei* (Figure 4a) and *M. kandleri* (Figure 4b) markedly resemble the 532 nm‐excited Raman spectrum of cyanocobalamine in aqueous solution (Figure 4c). The high spectral resemblance among the three spectra compared in Figure 4, together with the well‐established fact that methanogens produce factor III and/or pseudo‐vitamin B_12_,^15^ lead us to attribute the observed difference spectra (i.e., the photobleaching component) to cobamides. The in vitro UV/Vis absorption spectra of factor III in the literature,^38^ as well as that of cyanocobalamin we measured (Figure 4c, inset), ensure that 532‐nm excitation did elicit resonance Raman spectra of cobamides.

The resonance Raman bands of cobamides are mainly associated with corrin ring vibrational modes. The type of the upper and lower ligands (see Figure 1) is known to have little effect on the peak positions of those bands.^27^, ^31^, ^32^, ^39^ According to recent papers by Machalska et al.,^31^, ^32^ the tentative assignments of prominent resonance Raman bands of cobamides are given as follows. The most intense band at ∼1500 cm^−1^ can be assigned to the C=C and C=N stretching with some contributions from the CH, CH_2_, and CH_3_ bending modes. The Raman bands at ∼1165 and ∼1200 cm^−1^ are assignable to the C–C stretching of the corrin ring plus the CH bending, CH_2_ twisting, and CH_3_ rocking modes. Additionally, the C–N stretching mode may also contribute to the band at ∼1165 cm^−1^. In *M. kandleri*, these two bands are observed with nearly equal amplitudes (Figure 4b). In contrast, in *M. mazei*, only the 1163 cm^−1^ band is clearly seen and the 1200 cm^−1^ band is barely distinguishable from noise (Figure 4a).

There are several weak features at 750, 1315, and 1580 cm^−1^ in the difference spectrum of *M. mazei* (Figure 4a) that are unlikely to originate from cobamides. Note that they are not observed in the difference spectrum of *M. kandler*i (Figure 4b). These bands are associated with cytochromes, because the reported 532 nm‐excited resonance Raman spectra of cytochromes (e.g., reduced cytochrome *c*) in fungi^36^, ^40^ exhibit intense bands exactly at these wavenumbers. Reduced cytochromes have another intense Raman band at ∼1128 cm^−1^, but it could be buried under the foot of the 1163 cm^−1^ band of cobamides. The above assignment is consistent with the fact that *M. mazei* possesses cytochromes but *M. kandleri* does not.^41^ Table 1 summarises the peak positions of the major Raman bands that are found in Figures 3 and 4a,b and the biomolecular components that are responsible for those bands.

**TABLE 1:** Peak positions of the major Raman bands observed in the four methanogens and their biomolecular assignments

**Wavenumber (cm^−1^)**	**Biomolecules**
419 (*M. barkeri*), 420 (*M. kandleri*), 422 (*M. mazei*), 427 (CNCbl^a^)	Cobamides^27^ ^,^ ^31^
632 (CNCbl), 633 (*M. mazei*), 634 (*M. kandleri*), 637 (*M. barkeri*)	Cobamides
724, 732 (*M. mazei*), 726 (*M. kandleri*), 728 (CNCbl), 730 (*M. barkeri*)	Cobamides
747 (*M. barkeri*), 750 (*M. mazei*)	Cytochromes^36^
845 (*M. barkeri*), 846 (CNCbl), 857 (*M. mazei*)	Cobamides
1003	Proteins^22^
1113 (*M. barkeri*)	Cytochromes
1163 (*M. mazei*), 1165 (*M. barkeri*, *M. kandleri*, CNCbl)	Cobamides
1199 (*M. mazei*, *M. barkeri*, *M. kandleri*), 1203 (CNCbl)	Cobamides
1315 (*M. mazei*)	Cytochromes
1343 (*M. mazei*), 1346 (*M. barkeri*), 1349 (CNCbl), 1350 (*M. jannaschii*), 1351 (*M. kandleri*)	Cobamides
1394 (*M. barkeri*), 1395 (CNCbl), 1396 (*M. mazei*)	Cobamides
1499 (CNCbl), 1500 (*M. jannaschii*), 1502 (*M. mazei*, *M. barkeri*), 1503 (*M. kandleri*)	Cobamides
1544 (CNCbl), 1545 (*M. barkeri*), 1547 (*M. mazei*)	Cobamides
1580 (*M. mazei*), 1588 (*M. barkeri*)	Cytochromes
1660	Proteins

^a^ CNCbl, cyanocobalamin.

**FIGURE 5:**
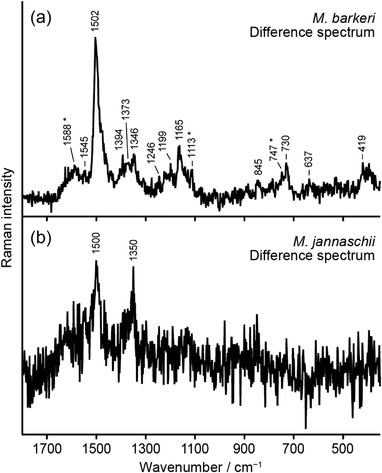
Difference Raman spectra of *M. barkeri* (a) and *M. jannaschii* (b), which were obtained by subtracting the last (135–150 s for *M. barkeri* and 60–75 s for *M. jannaschii*) spectrum from the first (0–15 s for both *M. barkeri* and *M. jannaschii*) spectrum recorded in consecutive measurements. The difference spectrum of *M. jannaschii* is the average over eight cells. Asterisks indicate Raman bands of cytochromes

Likewise, the photobleaching component of *M. barkeri* displays spectral features attributable to cobamides and cytochromes (Figure 5a). In *M. barkeri*, which belongs to the same genus *Methanosarcina* as *M. mazei*, the intensity of the 1165 cm^−1^ band is clearly stronger than that of the 1199 cm^−1^ band. The cytochrome bands of *M. barkeri* are not as prominent as those of *M. mazei*. The difference Raman spectrum of *M. jannaschii* (Figure 5b) suffers from the largest amount of noise among the four methanogens even after SVD denoising, but the Raman bands at ∼1350 and ∼1500 cm^−1^ can still be discerned. Preliminary attempts to detect the cobamide Raman signatures in cells of other methanogens, such as *Methanomassiliicoccus luminyensis* and *Methanocella paludicola*, were unsuccessful due to the insufficient degree of intracellular production and accumulation of cobamides and the small size of the cells.

## 4 DISCUSSION

Resonance Raman analysis has successfully detected cobamides in the methanogens *M. mazei*, *M. barkeri*, *M. kandleri*, and *M. jannaschii*. We examined a few nonmethanogenic archaea, but their spectra showed only cytochrome bands and no cobamide bands to any measurable extent (see Figure S1). High production of cobamides has been revealed not only in methanogens but also in multiple bacterial species such as propionic acid‐producing bacteria^14^; therefore, this method can also be applied directly to those bacterial strains. Although previous studies have shown using bulk analysis the presence of cobamides in methanogens,^17^, ^42^ this is the first report, to the best of our knowledge, on cobamide detection in microbial cells (not just methanogens). Single‐cell analysis enabled by resonance Raman microspectroscopy (as for the cases of *M. kandleri* and *M. jannaschii*) will be useful not only for understanding the heterogeneity in the cobamide production within a microbial population but also for identifying methanogenic cells that exhibit high metabolic activity. Serrano et al. have reported the single‐cell Raman spectra of some methanogens including *M. mazei*.^43^, ^44^ Their excitation wavelength is the same (i.e., 532 nm) as that used in the present study; the exposure time and laser power are comparable, too. Nevertheless, the cobamide bands were not detected in their work. We noticed that unlike our experiments, the researchers performed prolonged centrifugation during the sample preparation and air‐drying of the cell sample on a slide glass,^43^, ^44^ but the reason for the discrepancy between our results and theirs is unclear.

We attribute the difference in the relative intensity of the ∼1165 and ∼1200 cm^−1^ bands between *M. mazei* and *M. kandleri* (Figures 4a, 4b) to the difference in the upper axial ligand (the so‐called sixth ligand; see Figure 1). Accumulated resonance Raman spectra of cobalamines (vitamin B_12_ derivatives) all indicate that the corrin‐ring frequencies are surprisingly similar no matter what the axial ligand^27^, ^31^, ^32^, ^39^ or the oxidation state^27^, ^30^ of the cobalt atom (Co(II) or Co(III)) is. Close inspection of the spectra, however, reveals that the relative intensities of some minor bands differ substantially among cobalamines. Salama and Spiro have reported—but not discussed—that coenzyme B_12_, which possesses the 5′‐deoxyadenosyl group as the upper ligand (see Figure 1), exhibits a much reduced intensity for the 1200 cm^−1^ band compared to the 1165 cm^−1^ band.^27^ This feature agrees fairly well with the aforementioned pattern observed in our difference spectra of *M. mazei* (Figure 4a) and *M. barkeri* (Figure 5a). Note that other cobalamines including methylcobalamine (R_1_ = CH_3_) and hydroxocobalamine (R_1_ = OH) give the two bands of nearly equal intensities,^27^, ^31^ as in the case of cyanocobalamine (Figure 4c) and of *M. kandleri* (Figure 4b). In addition to the relative intensities of these two bands, the spectral patterns around 730 cm^−1^ and 1340–1400 cm^−1^ in Figure 4a are also consistent with coenzyme B_12_. Taken together, the cobamide we detected in *M. mazei* is most likely factor III or pseudo‐vitamin B_12_ with the 5′‐deoxyadenosyl group being the upper ligand. These cobamides are presumably involved in DNA synthesis and other cellular reactions rather than methane synthesis.

We cannot conclude, at this stage, whether factor III or pseudo‐vitamine B_12_ is dominant in *M. mazei* cells. Some methanogens in the class *Methanomicrobia* including *Methanosarcina barkeri* ^38^ have been shown to have factor III as a predominant cobamide compound, but unfortunately, *M. mazei* has not been examined.^42^ There is currently no report in the literature on the cobamide(s) produced by *M. kandleri*. Further biochemical analysis will be required to fully identify the cobamides detected in the methanogenic cells. The product(s) of photodegradation of cobamides^45^, ^46^, ^47^ in methanogenic cells also remain to be identified.

Besides cobamides, various unusual cofactors have been found in methanogens.^15^ The nickel‐corrinoid complex known as F_430_ is a unique cofactor of methanogens and plays an important role in methane synthesis.^15^ F_430_ has a corrin ring like cobamides, but its Raman spectral pattern is different from that of cobamides^48^ and so is the absorption spectrum.^49^ The UV–Vis spectrum of F_430_ shows no appreciable absorption at around 532 nm, which agrees with the absence of the F_430_ Raman bands in our spectra.

The ability of the resonance Raman method to detect another important biomolecular species, cytochromes, together with cobamides is noteworthy. In general, methanogens can be divided into two groups depending on whether they possess cytochromes or not for methane synthesis. Most of cytochrome‐containing methanogens can utilize a more variety of substrates such as acetate and methylamines to produce methane^41^ than non‐cytochrome‐containing methanogens. *M. mazei* and *M. barkeri* belong to the former group, a knowledge that has allowed us to attribute the non‐cobamide resonance Raman bands at 750, 1315, and 1580 cm^−1^ (asterisked bands in Figures 4a and 5a) to cytochromes. This result suggests that the resonance Raman analysis using 532‐nm excitation can distinguish between cytochrome‐containing and non‐cytochrome‐containing methanogenic cells, provided that the cells produce enough cobamides to be detectable and that the laser intensity and exposure time are carefully adjusted to minimize the photobleaching effect.

## 5 CONCLUSION

In the present study, we have demonstrated sensitive detection of intracellular cobamides in four methanogenic (*M. mazei*, *M. barkeri*, *M. kandleri*, and *M. jannaschii*) cells using resonance Raman microspectroscopy. Our results show that cobamide detection is feasible regardless of whether methanogens possess cytochromes or not and from where they were isolated (i.e., rice fields or hyperthermal deep‐sea vents). This method will thus pave the way to analyzing the cell distribution and physiological activity of methanogens relating to the greenhouse gas production and extreme microbial ecosystems. Further studies should extend it to include more diverse methanogenic species that inhabit a wide range of natural, extreme, and industrial environments.

## AUTHOR CONTRIBUTIONS

N.K. and S.S. designed research, interpreted data, and wrote the manuscript. N.K. performed research and analyzed data. S.K., T.I., and M.O. provided microbial samples.

## CONFLICT OF INTEREST

The authors declare no conflict of interest.

## Supporting information

SUPPORTING INFORMATION

## Data Availability

Data are available from the corresponding author upon reasonable request.
