# Supplementary material for: Resonance Raman analysis of intracellular vitamin B12 analogs in methanogenic archaea
Source: Anal Sci Adv. 2022 Jan 9;3(5-6):165–73. doi: 10.1002/ansa.202100042 (PMC10989573; doi:10.1002/ansa.202100042)
Supplement: Supplementary file 1 — SUPPORTING INFORMATION [file ANSA-3-165-s001.pdf]

Supporting Information

Resonance Raman analysis of intracellular vitamin B_{12} analogues in methanogenic archaea

Nanako Kanno,[1] Shingo Kato,[2] Takashi Itoh,[2] Moriya Ohkuma,[2] and Shinsuke Shigeto[1]*

[1]Department of Chemistry, School of Science, Kwansei Gakuin University, 2-1 Gakuen, Sanda, Hyogo 669-1337, Japan.
[2]Japan Collection of Microorganisms, RIKEN BioResource Research Center, 3-1-1 Koyadai, Tsukuba, Ibaraki 305-0074, Japan.

*Corresponding author.
Email: shigeto@kwansei.ac.jp

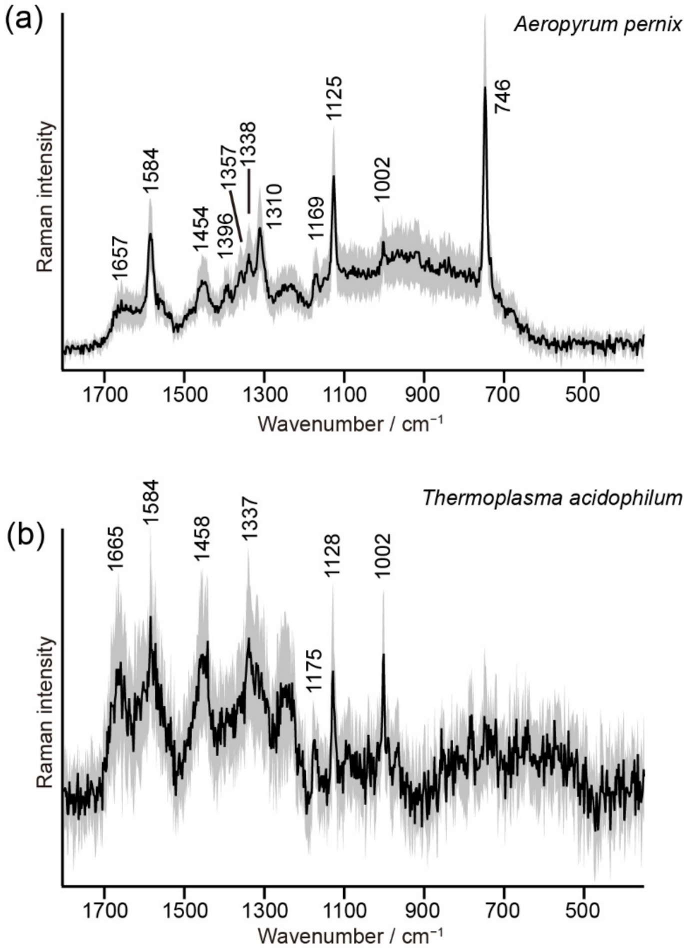

Figure S1. Raman spectra of non-methanogenic archaea *Aeropyrum pernix* JCM 9820^{T} (a) and *Thermoplasma acidophilum* JCM 9062^{T} (b). Both archaea were obtained from Japan Collection of Microorganisms (JCM). Cell cultures were used as received from JCM for Raman measurements within 7 days of shipping. Cells of *A. pernix* were washed three times with PBS containing 3% NaCl by centrifugation (10,000 × *g*, 1 min) at room temperature and then resuspended in PBS with 3% NaCl. Cells of *T. acidophilum* were washed three times with PBS (pH 5.0) by centrifugation (10,000 × *g*, 1 min) at room temperature and then resuspended in PBS (pH 5.0). For each single-cell Raman spectrum, the PBS spectrum (average of 10 spectra) was subtracted, followed by baseline correction using second-order (*A. pernix*) or fifth-order (*T. acidophilum*) polynomial fitting. The resulting Raman spectra of 10 cells were then averaged and shown here. The shaded area represents the 1σ error envelope.
